# Reconversion of Parahydrogen Gas in Surfactant-Coated Glass NMR Tubes

**DOI:** 10.3390/molecules28052329

**Published:** 2023-03-02

**Authors:** Robert V. Chimenti, James Daley, James Sack, Jennifer Necsutu, Nicholas Whiting

**Affiliations:** 1 Department of Physics & Astronomy, Rowan University, Glassboro, NJ 08028, USA; 2 Advanced Materials & Manufacturing Institute, Rowan University, Glassboro, NJ 08028, USA; 3 Department of Chemistry & Biochemistry, Rowan University, Glassboro, NJ 08028, USA; 4 Department of Biological & Biomedical Sciences, Rowan University, Glassboro, NJ 08028, USA

**Keywords:** parahydrogen, orthohydrogen conversion, glass surfactant, Raman spectroscopy

## Abstract

The application of parahydrogen gas to enhance the magnetic resonance signals of a diversity of chemical species has increased substantially in the last decade. Parahydrogen is prepared by lowering the temperature of hydrogen gas in the presence of a catalyst; this enriches the para spin isomer beyond its normal abundance of 25% at thermal equilibrium. Indeed, parahydrogen fractions that approach unity can be attained at sufficiently low temperatures. Once enriched, the gas will revert to its normal isomeric ratio over the course of hours or days, depending on the surface chemistry of the storage container. Although parahydrogen enjoys long lifetimes when stored in aluminum cylinders, the reconversion rate is significantly faster in glass containers due to the prevalence of paramagnetic impurities that are present within the glass. This accelerated reconversion is especially relevant for nuclear magnetic resonance (NMR) applications due to the use of glass sample tubes. The work presented here investigates how the parahydrogen reconversion rate is affected by surfactant coatings on the inside surface of valved borosilicate glass NMR sample tubes. Raman spectroscopy was used to monitor changes to the ratio of the (J: 0 → 2) vs. (J: 1 → 3) transitions that are indicative of the para and ortho spin isomers, respectively. Nine different silane and siloxane-based surfactants of varying size and branching structures were examined, and most increased the parahydrogen reconversion time by 1.5×–2× compared with equivalent sample tubes that were not treated with surfactant. This includes expanding the pH_2_ reconversion time from 280 min in a control sample to 625 min when the same tube is coated with (3-Glycidoxypropyl)trimethoxysilane.

## 1. Introduction

Hydrogen gas possesses two distinct spin isomers—*para* (pH_2_) and *ortho* (oH_2_) [1]. Parahydrogen occupies the nuclear singlet state:

(1) $$│S_{0}\rangle =\frac{│\alpha \beta \rangle -│\beta \alpha \rangle }{\sqrt{2}}$$

with the two hydrogen nuclear spin states (*α*, *β*) configured as anti-aligned [2]. Although the wavefunction of molecular H_2_ consists of electronic, vibrational, rotational, translational, and nuclear spin components, only the rotational and nuclear spin contributions are affected by the exchange of nuclei [3,4]. Furthermore, because the total wavefunction of a homonuclear diatom consisting of two fermions (I = ½ for hydrogen) must be antisymmetric with respect to exchange (to satisfy the Pauli exclusion principle) [5], the two spin isomers of H_2_ will have either a symmetric rotational wavefunction coupled with an antisymmetric nuclear spin wavefunction, or vice versa [5,6]. For parahydrogen, this means that the anti-aligned nuclear spins experience symmetrical rotational states (e.g., J: 0, 2, 4…), whereas the molecule possesses no net nuclear spin (i.e., silent to magnetic resonance detection). Conversely, orthohydrogen occupies the nuclear triplet spin state:

(2) $$│T_{+}\rangle =│\alpha \alpha \rangle$$

(3) $$│T_{0}\rangle =\frac{│\alpha \beta \rangle +│\beta \alpha \rangle }{\sqrt{2}}$$

(4) $$│T_{-}\rangle =│\beta \beta \rangle$$

with three degenerate spin states (net nuclear spin: I = 1) that are symmetric under permutation and antisymmetric rotational quantum numbers (e.g., J: 1, 3, 5…) [2]. As evident by the rotational states, pH_2_ occupies the lower energy level and is the isomer of preferred abundance at low temperatures. Indeed, the difference in energy between the ground states of the para (J = 0) and ortho (J = 1) spin isomers has been reported to range between 169.7 K and 175.2 K [5,7,8], depending on the determination of the rotational constant.

The relative abundance of each spin isomer of H_2_ is dependent on the temperature of the gas and can be calculated using Boltzmann statistics [9]. When thermally equilibrated with room temperature (~295 K), the normal isomeric ratio of hydrogen gas (‘normal hydrogen’ or nH_2_) reflects the 1:3 distribution of the four total spin states for the two isomers: 25% pH_2_ vs. 75% oH_2_. Simply lowering the temperature of the gas increases the relative abundance of pH_2_; common pH_2_ fractions that are used for magnetic resonance applications range from ~50% pH_2_ at 77 K [10,11,12] to ~100% pH_2_ at 20 K [13,14,15], along with varying isomeric fractions at temperatures in between [16,17]. Because the interconversion between triplet and singlet states is quantum mechanically forbidden, converting between spin states proceeds slowly and is typically accelerated by exposing the gas to a paramagnetic catalysts (e.g., HFeO_2_) at the low temperature [18]. Parahydrogen generators can be lab constructed [12] or commercially procured and provide a constant flow of pH_2_-enriched gas to be used immediately for enhanced magnetic resonance (MR) experiments or stored for future use [19,20].

Although parahydrogen gas does not provide any magnetic resonance signal itself (each anti-aligned spin-½ hydrogen nuclei cancels the other, leaving the net nuclear spin as I = 0 for the diatom), it possesses near-unity spin order that can be transferred to nuclei in other chemical species, thus enhancing their MR signals by several orders of magnitude. The two common pathways for achieving this are termed parahydrogen induced polarization (PHIP)—a hydrogenative process that results in a new chemical species [21]—and signal amplification by reversible exchange (SABRE)—a non-hydrogenative pathway that does not chemically modify the substrate [22,23]. Importantly, PHIP and SABRE allow the high nuclear spin order of pH_2_ to be transferred to nuclei with low gyromagnetic ratios or isotopic abundances (e.g., ^13^C, ^15^N, etc.) in other molecules of interest [24,25,26,27] to allow their facile detection, including in dilute concentrations and at low magnetic fields. Using these methods, the MR signals for a range of biologically relevant small molecules have been enhanced, including in in vitro [28] and in vivo [29] scenarios. Furthermore, parahydrogen-based techniques benefit from low cost, ease-of-use, and fast cycle times compared with other MR signal enhancement approaches, such as spin-exchange optical pumping [30] and dynamic nuclear polarization [31].

After the pH_2_-enriched gas is generated, it retains its para spin abundance even after warming to room temperature. Over time, the gas slowly reverts to its normal isomeric fraction of 25% pH_2_ [32]. The rate of this reconversion (*T*_ρ_) is variable and can range from minutes (in the presence of isotropic paramagnetic impurities, such as gaseous O_2_) to hours (e.g., inside glass containers) to even weeks (e.g., in pressurized aluminum cylinders [33]). This long reconversion time is attributed to the forbidden singlet → triplet transition, and the absence of any paramagnetic species mitigates the speed of the conversion [34]. The reported use of pH_2_ to enhance nuclear magnetic resonance (NMR) signals has increased significantly since the introduction of SABRE in 2009 [22]. NMR studies typically use borosilicate glass tubes to complete liquid and gas-phase experiments. Borosilicate glass often contains a heterogeneous distribution of paramagnetic impurities in the form of Fe^2+^, Fe^3+^, Co, and Cr, etc. [35,36]. NMR sample tubes constructed of quartz or sapphire are also available and contain fewer impurities, albeit at a higher price that may not be conducive to widespread adoption. Indeed, the presence of paramagnetic impurities in glass also affects other hyperpolarized MR techniques, such as spin-exchange optical pumping of ^3^He [37] or ^129^Xe [38]. Although pH_2_ may not directly contact these paramagnetic centers in borosilicate glass, their proximity to the glass surface is sufficient to catalyze the reversion of pH_2_ to nH_2_ (via dipolar effects) at a higher rate than when stored in containers of differing surface composition (e.g., aluminum). Typical *T*_ρ_ values for pH_2_ in glass NMR tubes have been measured at ~850 min [14,33]; however, larger and smaller *T*_ρ_ values have also been reported [11,39,40] under different experimental conditions. Not only does pH_2_ revert faster in glass NMR tubes (compared with aluminum bottles) but the variability of this reconversion depends on the individual tube; for example, Parrot et al. reported a ~5-fold difference in *T*_ρ_ for two off-the shelf NMR tubes from the same manufacturer [40]. The reversion of pH_2_ to nH_2_ is further confounded by the potential presence of O_2_ gas [41] within the NMR tube due to incomplete evacuation or a leaking valve [39].

Although the recent interest in pH_2_ has focused on its ability to enhance the NMR signals of other molecules of interest [42], the different rotational levels occupied by the para and ortho spin isomers provide a convenient method of observing the pH_2_ enrichment level using Raman spectroscopy (H_2_ rotational constant is reported to be between 84.8 K and 87.6 K [7]). The integrals of the first-order transitions of the lower-energy pH_2_ Raman peak (e.g., 355 cm^−1^) and higher-energy oH_2_ peak (e.g., 586 cm^−1^) can be compared in real time to determine the relative abundances of the two spin isomers. This internal comparison mitigates the need for a reference standard to determine the enrichment of pH_2_ in the gas sample, and Raman spectroscopy is significantly faster than NMR detection of the gas (remembering that the pH_2_ fraction does not provide an MR signal and must be deduced from changes to the oH_2_ MR signal over time) [19]. Furthermore, Raman spectroscopy can probe the gas in situ while stored within a glass NMR tube, with minimal interference from the glass container. Raman spectroscopy has been used previously to interrogate parahydrogen systems [43] across a range of analysis conditions, including the use of Raman microscopes [34,44], specialized gas cells with relatively high pressures (e.g., 345 bar) [45,46], or long integration times (e.g., 20 min per scan) [47]. Parrott et al. recently reported in situ measurements of pH_2_ enrichments from a parahydrogen generator using an in-line flow tube and non-contact backscattered Raman collection at lower pressures (e.g., 4 bar) and short integration times (e.g., 30 s) [40]; their study focused on characterizing and optimizing the production of a newly constructed pH_2_ generator for MR applications.

**Figure 1:**
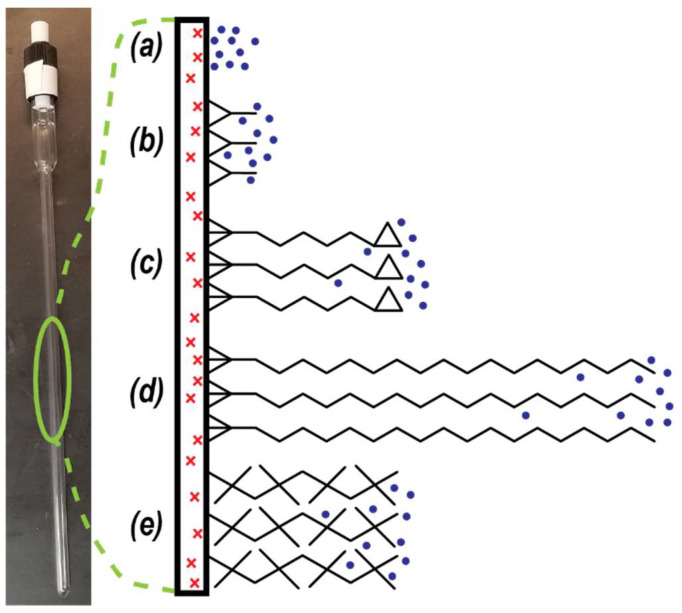
Schematic of different surfactant coatings on the inside glass surface of a valved NMR tube. Red ‘x’ denotes paramagnetic impurity within glass surface. Blue dots represent pH_2_ molecules in the gas phase. Black chemical structures are different surfactants: (**a**) bare glass, (**b**) DCS, (**c**) GOTTS, (**d**) OTS, and (**e**) DOTTS. See Table 1 for full names and chemical formulas.

In this work, Raman spectroscopy was used to probe the reconversion of pH_2_ to nH_2_ in parahydrogen-enriched gas samples that were stored in a valved glass NMR tube. Nine different common surfactants were used to coat the inside surface of the tube to serve as a barrier to physically distance the pH_2_-enriched gas from the paramagnetic impurities within the glass (Figure 1). These surfactants varied in chemical composition, molecular length, and branching structure. Rotational Raman spectroscopy was performed on the gas samples directly through the NMR tubes, and changes to the ratio of the 586 cm^−1^ (oH_2_) vs. 355 cm^−1^ (pH_2_) peaks were plotted over time to measure the para-to-ortho conversion rate for H_2_ gas while in the presence of each surfactant. It was found that the addition of surfactants extended the pH_2_ reconversion time by a factor of 1.5–2× compared with untreated NMR tubes. This research is of particular importance to scientists who wish to maximize the lifetime of pH_2_ gas for storage in glass containers (including NMR applications).

## 2. Experimental

### 2.1. Parahydrogen Generator

**Figure 2:**
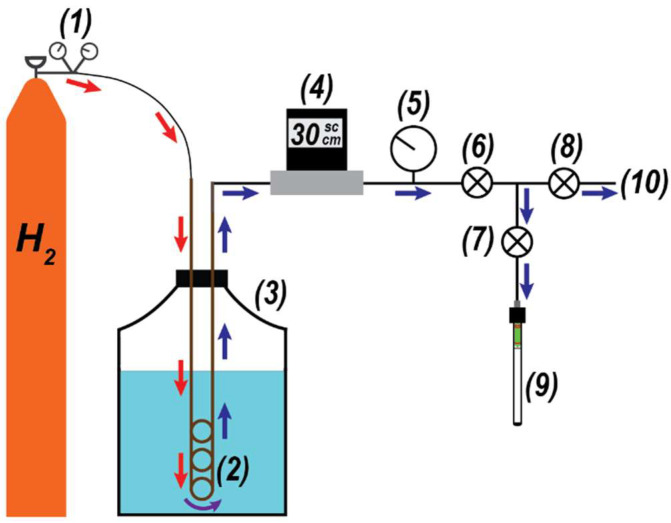
Parahydrogen generator schematic. H_2_ gas (1) is directed into the conversion coil (2) submerged in a 10 L liquid nitrogen Dewar (3). The flow is regulated by an in-line controller (4), and a sample-side pressure gauge (5) is used for loading. Valves (6–8) are used to either direct the pH_2_-enriched gas to a pre-evacuated, valved NMR tube (9) or exhaust the gas to the fume hood (10).

This study utilized a laboratory-constructed pH_2_ generator that consistently produced parahydrogen enrichments of ~50%; Figure 2 provides a general overview of the device. The generator used high-purity H_2_ gas (99.999%) that was pressure-regulated at ~3.45 bar; this gas was directed to a conversion coil that was submerged in liquid nitrogen. The conversion coil was a ¼″ outer diameter copper tube (3 m length) that was bent into a multicoil. Inside of the bottom loops of the conversion coil, ~34 g of HFeO_2_ catalyst (Aldrich, St. Louis, MO, USA; 371254-250G; 30–50 mesh) was placed in the flow path of the H_2_ gas. The catalyst, which facilitated the conversion from oH_2_ to pH_2_, was retained in the coil using cotton wads. A calibrated flow controller (Sierra Instruments, Monterey, CA, USA) maintained the H_2_ flow rate at 30 standard cubic centimeters per minute (sccm) during experiments, and ¼″ PTFE tubing was used to move the gas between the H_2_ cylinder, conversion coil, flow controller, and manifold. When beginning an experiment, H_2_ gas was purged through the conversion coil (60 sccm) at room temperature for 30 min to remove residual atmosphere from the coil. Afterwards, the coil was submerged into the 10 L liquid nitrogen Dewar (MVE Lab10) and the H_2_ flow rate was decreased to 30 sccm. Gas sample collection would begin one hour after submersion of the conversion coil into the liquid nitrogen Dewar. Parahydrogen-enriched gas samples were collected in pre-evacuated (13.3 Pa) valved NMR tubes (Wilmad Labglass, Vineland, NJ, USA; 524-QPV-7), with a total of ~3.04 bar of gas loaded into each tube (~2.07 bar above atmosphere). Any uncollected H_2_ gas was exhausted to the laboratory fume hood.

### 2.2. Surfactants

Nine different commercially sourced surfactants were used to coat the inside surface of a valved NMR tube for the Raman studies in this work. These surfactants were mostly silane based, along with those possessing siloxane, laurate, and proprietary formulas. The surfactants were generically categorized by the size and shape of their chemical linkages: short (<5 links), medium (~10 links), long (~20+ links), or branched. Table 1 highlights the chemical information for the surfactants. Starting with a clean and dry NMR tube, a solution of 20% surfactant (by volume) in heptane was added to the tube and allowed to rest for 20 min. The solution was then decanted and the tube was rinsed in heptane (3×) and allowed to dry at room temperature overnight. Two of the surfactants received modifications to this protocol: Sigmacote (a proprietary solution of chlorinated organopolysiloxane in heptane; Sigma Aldrich, Burlington, MA, USA) was used as received without further dilution. Tween-80 (polyoxyethylene sorbitan monolaureate; MP Biomedicals, Santa Ana, CA, USA) was diluted and rinsed in ethanol (20% *v*/*v*) instead of heptane. Prior to loading the pH_2_, the tube was evacuated to ~13.3 Pa and attached to the gas loading manifold (Figure 2). Following gas loading and Raman spectroscopy, the tube was stripped of surfactant by filling with a base solution (supersaturated solution of NaOH in methanol) overnight. Afterwards, the base solution was decanted, the NMR tube was rinsed with methanol (3×), filled with methanol, and suspended in a heated ultrasonic bath for 40 min at 50 °C, followed by a methanol rinse. The tube received a final rinse with heptane and was allowed to dry overnight (at room temperature) before repeating the procedure to add a new surfactant. A single NMR tube was used to control for variability in paramagnetic impurities in the glassware, integrity of the valve, and background Raman signal. The order of surfactant choice was random, and no hysteresis was noticed in the *T*_ρ_ data. A water bead test established adequate removal of old surfactant between experiments; this was also confirmed in select cases using Raman measurements (Supplemental Materials Figure S5).

### 2.3. Raman Spectroscopy

**Figure 3:**
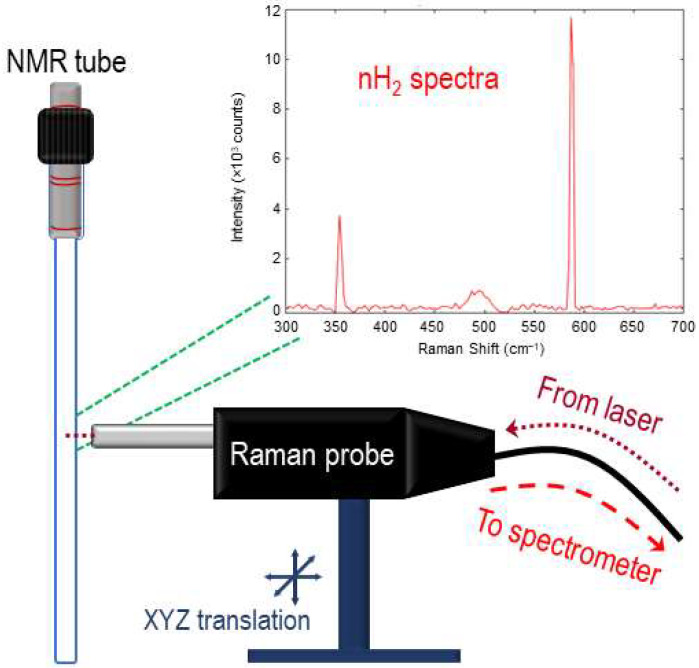
Raman collection setup. Bifurcated fiber-optic-coupled Raman probe on XYZ translational stage is directed to the center volume of a valved NMR tube, allowing for the collection of rotational Raman spectroscopy of the isomerically evolving H_2_ gas.

All Raman spectra were collected using a volume Bragg grating stabilized 785 nm diode laser (Innovative Photonic Solutions, Plainsboro, NJ, USA), which was internally coupled to a multi-mode fiber-optic with FC/PC termination. The laser was joined to the excitation leg of a bifurcated fiber-optic Raman probe (Innovative Photonic Solutions) and aligned to the center of the NMR tube using a homebuilt XYZ micro-positioning stage. The probe delivered ~290 mW of laser power to the sample in a ~100 μm diameter spot directed to the midsection of the NMR tube. The collection leg of the probe was coupled to a high-throughput transmission (f/1.6) spectrograph (Ibsen Photonics, Farum, Denmark). Raman signals were collected using a deep depleted back-illuminated CCD camera (Andor Instruments, Belfast, UK) that was thermoelectrically cooled to −60 °C. A schematic of the Raman collection setup is found in Figure 3. Integration times of 30 s were used for data collection, and spectra were collected continuously during the course of the experiment (e.g., 50 h). The spectral data were analyzed using in-house MATLAB code, which included an open-source adaptive iteratively reweighted penalized least squares (airPLS) algorithm for baseline correction.

## 3. Results and Discussion

### 3.1. Measuring T_ρ_

**Figure 4:**
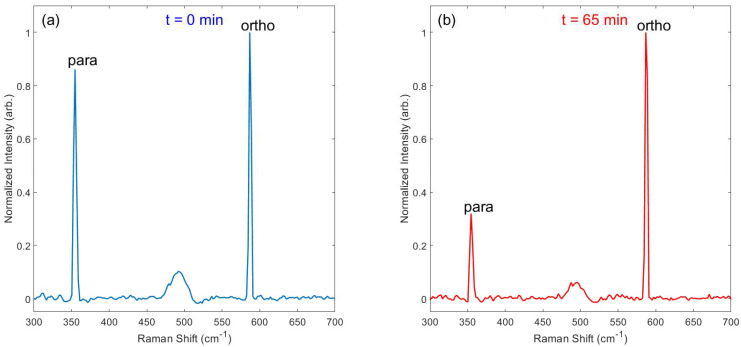
Example Raman spectra of the *J*: 0 → 2 ‘para’ spin peak (355 cm^−1^) and *J*: 1 → 3 ‘ortho’ spin peak (586 cm^−1^), taken both (**a**) immediately after loading the pH_2_-enriched gas into the valved NMR tube and (**b**) after 65 h had elapsed. The relative abundances of the two spin isomers can be determined from their relative peaks (e.g., the pH_2_ fraction is nearly 50% at *t* = 0 and reverts to the thermal equilibrium value of 25% within 65 h). See Supplemental Figure S2 for a version of this plot with the peaks overlaid, demonstrating consistent shift and linewidth between time points.

Immediately after loading pH_2_-enriched gas (~3.04 bar) into the evacuated NMR tube, Raman spectra were collected continuously (30 s integration time per acquisition) over the course of several hours. Figure 4 provides an example of spectra obtained on the J: 0 → 2 transition at 355 cm^−1^ for the para spin isomer and J: 1 → 3 transition at 586 cm^−1^ for the ortho spin isomer, both immediately following pH_2_-enriched gas loading and after 65 h had elapsed. At the beginning of the experiment, the para and ortho spin isomers are nearly equal in population (e.g., ~50% pH_2_), leading to similarly sized Raman peaks. At the end of the experiment, the characteristic 3:1 ratio of ortho-to-para spins is evident by the relative integrals of the Raman peaks at thermal equilibrium. Spectra were also observed for the higher energy J: 2 → 4 (para; 812 cm^−1^) and J: 3 → 5 (ortho; 1032 cm^−1^) transitions (Supplemental Figure S1). These higher-energy peaks were significantly weaker than the peaks at 355 cm^−1^ and 586 cm^−1^, and no other peaks were observed at transitions above 1032 cm^−1^ (e.g., vibrational levels).

**Figure 5:**
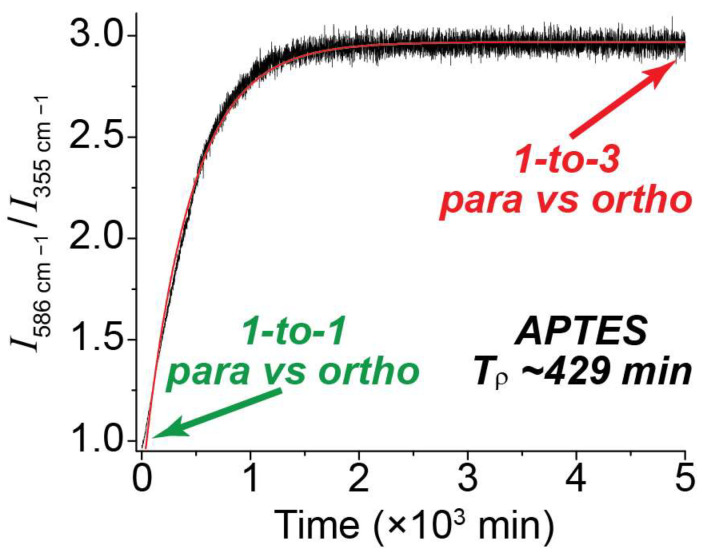
Plot comparing the ratio of integrals for the 586 cm^−1^ peak (*ortho*) vs. the 355 cm^−1^ peak (*para*) over time in a valved NMR tube coated with APTES. The initial 1-to-1 ratio denotes a 50/50 mixture of para and ortho spins, which slowly equilibrates to a 1-to-3 ratio of para vs. ortho upon reconversion.

In order to determine the para-to-ortho reconversion rate, the ratios of the 586 cm^−1^ vs. 355 cm^−1^ peak integrals were plotted over an extended timeline (Figure 5). At the start time, the ratio of the peaks was approximately 1-to-1, indicating a pH_2_ enrichment of ~50%. Over time, as pH_2_ converted to oH_2_, the ratio grew to approximately [40] 3-to-1; this is expected at thermal equilibrium and reflects the anticipated 75/25 fraction of ortho-para H_2_ at room temperature. This plot was repeated for all NMR tube surface conditions (Supplemental Figure S3), and the data was fitted to a monoexponential function to allow determination of *T*_ρ_. Given the significantly reduced intensity of the higher-order transitions (e.g., 812 cm^−1^ and 1032 cm^−1^) compared with the first-order peaks as well as the similarity of their observed populations, the J: 2 → 4 and J: 3 → 5 peaks were not factored into the ortho vs. para ratios described in this paper.

### 3.2. Comparing T_ρ_ across Surfactants

**Figure 6:**
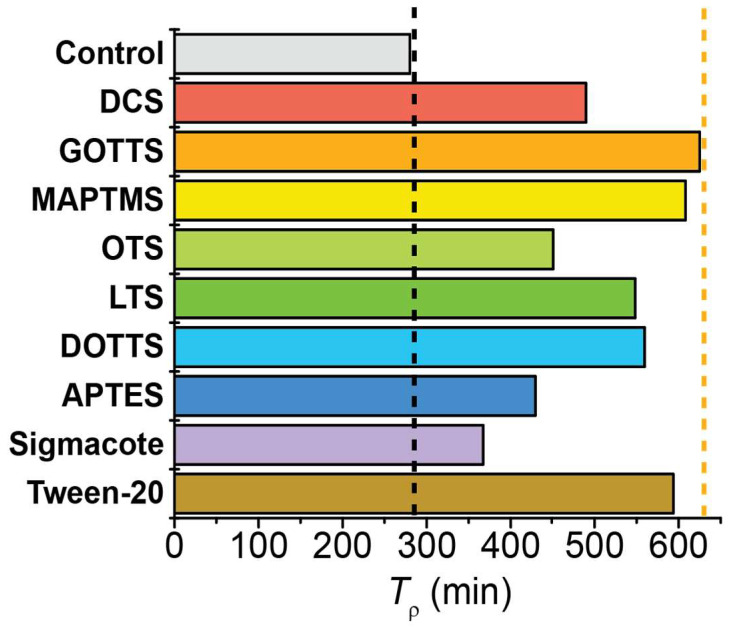
Plot comparing the pH_2_ *T*_ρ_ values for a valved NMR tube coated with different surfactants. All surfactants provided longer *T*_ρ_ values than the control (uncoated glass), with some doubling the control’s *T*_ρ_ value. Vertical black dashed line marks the *T*_ρ_ value of the control; vertical orange dashed line marks the longest *T*_ρ_ value observed. Supplemental Figure S4 plots this data grouped by surfactant category (e.g., small, medium, long, branched).

The *T*_ρ_ values calculated for each surface chemistry are compared in Figure 6. The control case was a bare, untreated NMR tube; this tube exhibited a *T*_ρ_ value of 280.2 min. The addition of surfactants resulted in variable increases to the *T*_ρ_ values; this ranged from a 31% increase for Sigmacote to a 123% increase for GOTTS. Despite testing surfactants of varying length and branching, there was no overarching dependence of *T*_ρ_ on the classification of surfactant. In general, the ‘medium length’ surfactants (~10 carbon atoms) exhibited the longest *T*_ρ_ values, with 625.0 min for GOTTS and 608.1 min for MAPTMS. However, the next several surfactants that provided the longest pH_2_ *T*_ρ_ values were of varying classification: Tween-20 (long, non-silane; 593.5 min), DOTTS (medium, branched; 559.4 min), and LTS (long; 548.4 min). The four surfactants with the least effect on *T*_ρ_ were DCS (short; 489.6 min), OTS (long; 450.8 min), APTES (short; 429.4 min), and Sigmacote (proprietary; 367.3 min). Although these four surfactant choices were deemed as the least effective for maximizing *T*_ρ_, it should be noted that they still increased the pH_2_ lifetime by 31–75% compared with the uncoated NMR tube.

### 3.3. Utilizing Surfactants for Hyperpolarized Media

The presence of paramagnetic impurities within glass has been a nuisance to various sectors of the hyperpolarized MR community over the last several decades. Spin-exchange optical pumping cells for enhancing the MR signals of noble gases (e.g., ^129^Xe) are often coated in a silanizing agent prior to loading with alkali metals in order to extend the polarized lifetime of the gases [48]. Indeed, ^3^He hyperpolarization for MRI applications and as neutron spin filters often utilizes a special alumina silicate glass (GE180) to minimize ^3^He permeability and spin relaxation during optical pumping and gas storage [49]. The ability to significantly extend the lifetime of pH_2_ gas when stored in glass vessels is of particular interest to the hyperpolarized MR community. The use of glass surfactants in valved NMR tubes can be particularly beneficial to pH_2_ experiments utilizing the “shake” method of introducing the gas to a liquid sample within the tube [22,42]. In this instance, pH_2_-enriched gas (e.g., 1–9 bar) is loaded into an evacuated valved NMR tube that already contains the degassed sample that is to be hyperpolarized. The sample tube is then physically agitated to introduce the pH_2_-enriched gas into the sample solution to participate in PHIP reactions or SABRE interactions, which results in enhanced MR signals of the substrate molecules. This method can be employed multiple times with a single pH_2_ gas loading, and the use of surfactants may extend the lifetime of the pH_2_ in the gas phase above the liquid sample (albeit with unknown potential contributions from vaporized solvents). Furthermore, recent work on hyperpolarizing gas-phase molecules (e.g., propane) [50] using parahydrogen may also benefit from the use of surfactants, with the added possibility of prolonging the hyperpolarized lifetime of the gas-phase substrate molecule (via minimizing wall-induced relaxation) in addition to the pH_2_ isomer. An additional potential benefit of incorporating surfactants is the ability for researchers to produce single aliquots of long-lasting pH_2_ pre-loaded into valved NMR tubes (also containing the hyperpolarizable sample) for NMR experiments at a different location (e.g., collaborator’s lab). This is in contrast to the common practice of researchers providing a pressurized aluminum cylinder of pH_2_-enriched gas that the collaborators must then introduce to the sample themselves (requiring additional skills and equipment).

### 3.4. Raman Measurements of Hydrogen Spin Isomers

Despite the regularity of using NMR measurements to quantify the pH_2_ fraction in valved NMR tubes, there are several distinct advantages of applying Raman spectroscopy instead. Because pH_2_ does not provide any NMR signal, a sample tube containing a mixture of pH_2_ and oH_2_ only provides a single ^1^H NMR peak (ascribed to the oH_2_). The signal acquisition then needs to be repeated over time to monitor the increasing intensity of that single peak (as pH_2_ converts to oH_2_) until the signal reaches equilibrium. Only after the sample has reverted to its normal isomeric ratio can the fraction of pH_2_ be indirectly measured from the corresponding increase in NMR signal intensity and knowledge of the Boltzmann distribution of spin states. Because the complete equilibration of spins may take days to conclude (e.g., 5 × *T*_ρ_), NMR is an inherently time-consuming approach to measure pH_2_ fractions in the gas phase. Conversely, Raman measurements can be advantageous because pH_2_ and oH_2_ each provide distinct rotational peaks, allowing the relative fractions of the two spin isomers can be determined within seconds from a single Raman spectrum. This functionality can also provide in-line quality assurance for pH_2_ generators [40]. Although the surfactant molecules used in this study are known to absorb mid-IR and far-IR light [51], this project did not observe absorption at the 785 nm laser line or nearby first-order Raman shifts.

### 3.5. Comparison with Previous T_ρ_ Measurements

Although this study has shown how glass surfactants can extend the *T*_ρ_ values of gas-phase parahydrogen, the actual values are less than other reported cases for parahydrogen reconversion in bare NMR tubes. For example, Feng et al. [33] used NMR to measure *T*_ρ_ in an uncoated low-pressure borosilicate NMR tube as ~850 min, which is ~3× longer than the *T*_ρ_ value measured in our control study. The most likely cause for this discrepancy is differences in the initial evacuation of the NMR tubes prior to gas loading. Whereas Feng et al. were able to evacuate their NMR tubes to less than 1.3 Pa, our system only allowed evacuation to ~13.3 Pa. This 10-fold difference in vacuum level allowed more gaseous oxygen to remain in our sample tubes with the parahydrogen; O_2_ has been previously shown [41] to hasten the reconversion of pH_2_ to nH_2_. Although the absolute numbers of *T*_ρ_ are lower in our study, the trend of improving *T*_ρ_ with the use of surfactant should provide even longer reconversion times in NMR tubes that are more extensively evacuated prior to gas loading. Indeed, if the same relative extension of pH_2_ lifetime is experienced in better-evacuated NMR tubes (such as [33]), then *T*_ρ_ values exceeding 30 h should be accessible in glass vessels. Additional future studies may monitor pH_2_ *T*_ρ_ in non-borosilicate NMR tubes, such as those constructed from quartz, zirconia, or sapphire, as well as monitor the effects of surfactant molecule polarity on the pH_2_ reconversion rate.

## 4. Conclusions

Extending the lifetime of pH_2_ in the gas phase is of particular importance to the hyperpolarized magnetic resonance community, as it expands the available timeline for enhancing the MR signals of various molecules of interest. This work demonstrates that the addition of silane-, siloxane-, and laureate-based surfactants to the inner glass surface of borosilicate NMR tubes increased the reconversion time of gaseous parahydrogen by factors ranging from 1.5–2× compared with an uncoated tube. This approach may be of particular interest for systems where pH_2_ gas is in contact with glass surfaces, such as for the “shake” method of PHIP and SABRE and hyperpolarizing gas-phase molecules using pH_2_. Furthermore, Raman spectroscopy provided a fast and simple in situ method to monitor the conversion of pH_2_-enriched gas to its normal isomeric abundance.

## Figures and Tables

**Table 1:** Chemical information regarding the surfactant molecules used in this study. Vendor information available in the Supplemental Materials Table S1.

Acronym	Chemical Name	Chemical Formula	Classification
Control	-----------	-------------	Control
DCS	Dichloromethylsilane	CH_3_SiHCl_2_	Short
GOTTS	(3-Glycidoxypropyl)trimethoxysilane	C_9_H_20_O_5_Si	Medium
MAPTMS	3-(Methacryloyloxy)propyltrimethoxysilane	C_10_H_20_O_5_Si	Medium
OTS	*n*-Octadecyltrichlorosilane	C_18_H_37_Cl_3_Si	Long
LTS	Lauryl triethoxysilane	C_18_H_40_O_3_Si	Long
DOTTS	1,7-Dichloro-octamethyltetrasiloxane	Cl[Si(CH_3_)_2_O]_3_Si(CH_3_)_2_Cl	Branched
APTES	(3-Aminopropyl)-triethoxysilane	H_2_N(CH_2_)_3_Si(OC_2_H_5_)_3_	Branched
Sigmacote	Sigmacote	Proprietary	Commercial
Tween-80	Polyoxyethylene sorbitan monolaurate	C_64_H_124_O_26_	Long, non-silane

## Data Availability

Data is available upon request to the corresponding author.

## Supplementary Materials

Supporting information can be downloaded at: https://www.mdpi.com/article/10.3390/molecules28052329/s1. Figure S1: Example extended Raman spectra of nH_2_ gas showing the first four rotational transitions: lower- energy J: 0 → 2 (para; 355 cm^−1^) and J: 1 → 3 (ortho; 586 cm^−1^) peaks, as well as higher-energy peaks at the J: 2 → 4 (para; 812 cm^−1^) and J: 3 → 5 (ortho; 1032 cm^−1^) transitions. Spectra collected using NMR tube coated with OTS surfactant at the time point of *t* = 3988 min after filling with pH_2_-enriched gas; Figure S2: Example Raman spectra of the J: 0 → 2 ‘para’ spin peak (355 cm^−1^) and J: 1 → 3 ‘ortho’ spin peak (586 cm^−1^), taken both immediately after loading the pH_2_- enriched gas into the valved NMR tube (blue solid line) and after 65 h had elapsed (red dashed line). This figure plots the same spectra that is available in Figure 4, and demonstrates consistent Raman shifts and linewidths for the peaks; Figure S3: Example *T*_ρ_ curves for the different experimental conditions. Ratio of the 586 cm^−1^ vs. 355 cm^−1^ peak integrals for each glass surface condition: (a) Control, (b) DCS, (c) GOTTS, (d) MAPTMS, (e) OTS, (f) LTS, (g) DOTTS, (h) APTES, (i) Sigmacote, and (j) Tween. Chemical information for each surfactant is available in Table 1 of the main document and Supplemental Table S1. Each dataset is fit to a single exponential decay function (red line) to extract the value of *T*_ρ_, along with the listed fit error. Please note the different x-axis scale for part (a); Figure S4: pH_2_ reconversion rate (*T*_ρ_) data from Figure 6 plotted with the surfactant types grouped. The x-axis denotes the surfactant type: control (C), short (S), medium (M), long (L), and branched (B); Figure S5: Comparison of the *I*_586 cm_^−1^ / *I*_355 cm_^−1^ time course measurements under three conditions: an NMR tube with no surfactant (Control 1; *T*_ρ_: 280 min; blue) followed by the same NMR tube coated with DOTTS surfactant (DOTTS; *T*_ρ_: 586 min; purple). The tube was then stripped of surfactant (following the protocol in Section 2.2 of the manuscript), and another time course measurement was completed (Control 2; *T*_ρ_: 271 min; red). This demonstrates that adequate surfactant removal between experiments was achieved; Table S1: Chemical and vendor information for surfactants used in this study, along with *T*_ρ_ tabulation (error indicative of equation fitting).
