# Supplementary material for: Reconversion of Parahydrogen Gas in Surfactant-Coated Glass NMR Tubes
Source: Molecules. 2023 Mar 2;28(5):2329. doi: 10.3390/molecules28052329 (PMC10004819; doi:10.3390/molecules28052329)
Supplement: Supplementary file 1 [file molecules-28-02329-s001.zip › molecules-2225853-supplementary.pdf]

Supplemental Document

Reconversion of Parahydrogen Gas in Surfactant-Coated Glass NMR Tubes

Robert V. Chimenti[1,2], James Daley[1,†], James Sack[1,†], Jennifer Necsutu[3,†], and Nicholas Whiting[1,4,*]

[1]Department of Physics & Astronomy, Rowan University, Glassboro, NJ 08028, USA

[2]Advanced Materials & Manufacturing Institute, Rowan University, Glassboro, NJ 08028, USA

[3]Department of Chemistry & Biochemistry, Rowan University, Glassboro, NJ 08028, USA

[4]Department of Biological & Biomedical Sciences, Rowan University, Glassboro, NJ 08028, USA

*Corresponding author:

Prof. Nicholas Whiting

Rowan University

101I Science Hall

201 Mullica Hill Dr.

Glassboro, NJ 08028

856-256-5436

whitingn@rowan.edu

[†]Undergraduate researcher

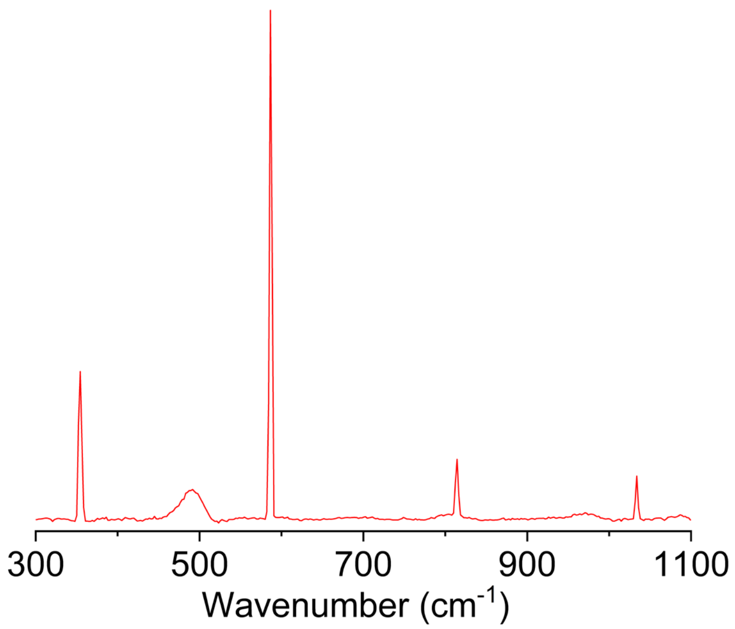

Supplemental Figure S1: Example extended Raman spectra of nH_2 gas showing the first four rotational transitions: lower-energy J: 0 → 2 (para; 355 cm^{-1}) and J: 1 → 3 (ortho; 586 cm^{-1}) peaks, as well as higher-energy peaks at the J: 2 → 4 (para; 812 cm^{-1}) and J: 3 → 5 (ortho; 1032 cm^{-1}) transitions. Spectra collected using NMR tube coated with OTS surfactant at the time point of t = 3,988 min after filling with pH_2-enriched gas.

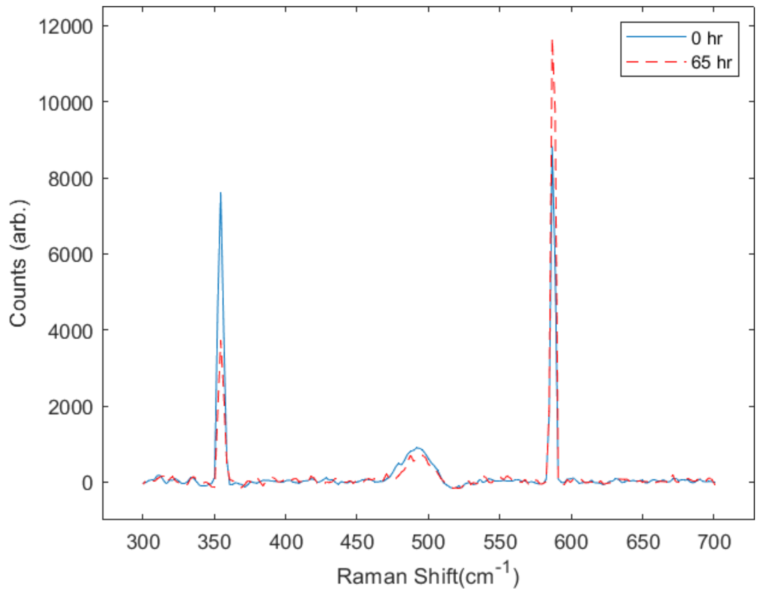

Supplemental Figure S2: Example Raman spectra of the *J*: 0 → 2 'para' spin peak (355 cm^{-1}) and *J*: 1 → 3 'ortho' spin peak (586 cm^{-1}), taken both immediately after loading the pH_2-enriched gas into the valved NMR tube (blue solid line) and after 65 hours had elapsed (red dashed line). This figure plots the same spectra that is available in Figure 4, and demonstrates consistent Raman shifts and linewidths for the peaks.

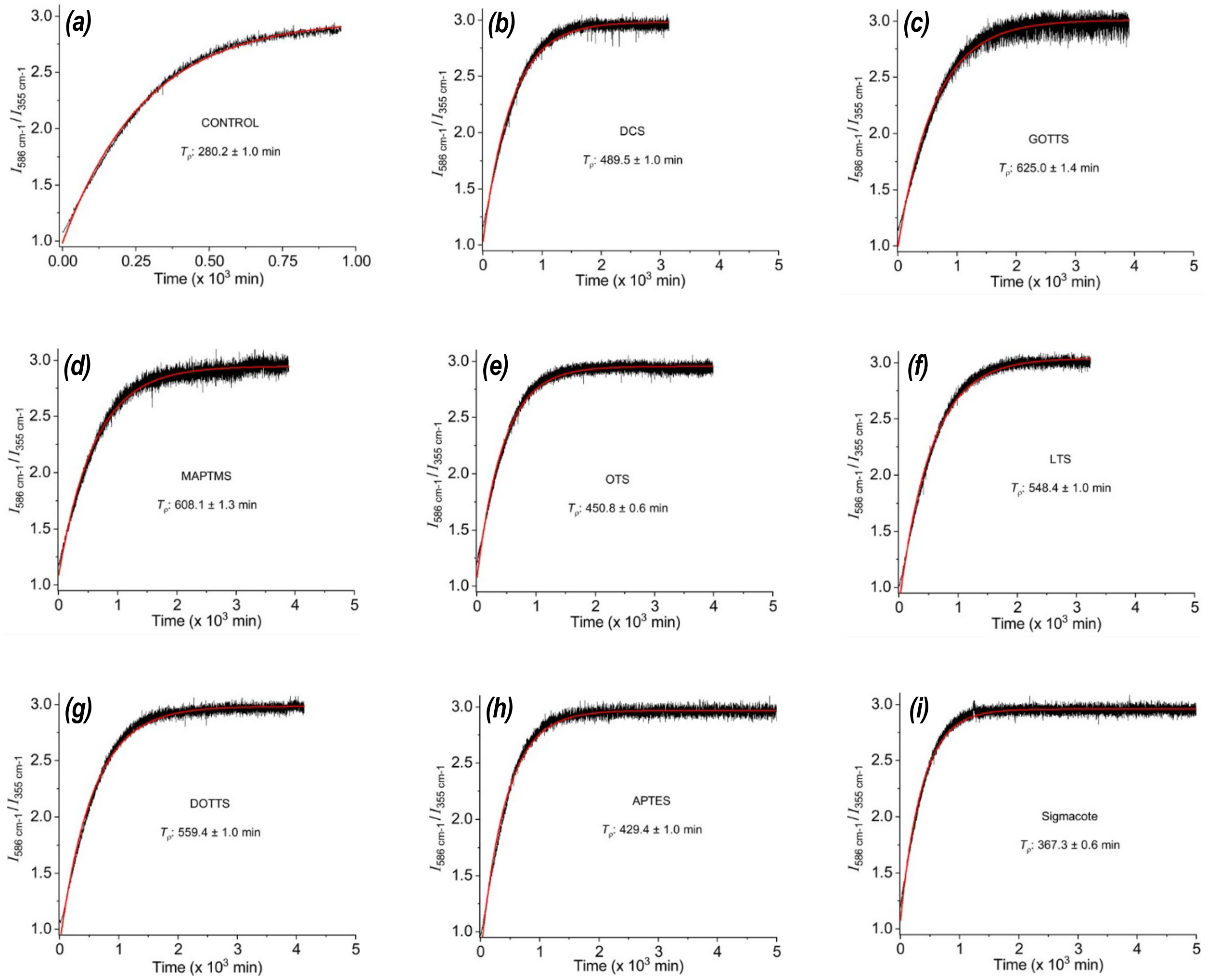

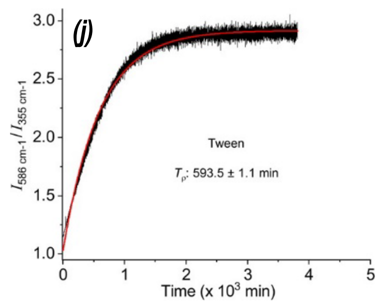

Supplemental Figure S3: Example T_ρ curves for the different experimental conditions. Ratio of the 586 cm^{-1} vs. 355 cm^{-1} peak integrals for each glass surface condition: ***(a)*** Control, ***(b)*** DCS, ***(c)*** GOTTS, ***(d)*** MAPTMS, ***(e)*** OTS, ***(f)*** LTS, ***(g)*** DOTTS, ***(h)*** APTES, ***(i)*** Sigmacote, and ***(j)*** Tween. Chemical information for each surfactant is available in Table 1 of the main document and Supplemental Table 1. Each dataset is fit to a single exponential decay function (red line) to extract the value of T_ρ, along with the listed fit error. Please note the different x-axis scale for part ***(a)***.

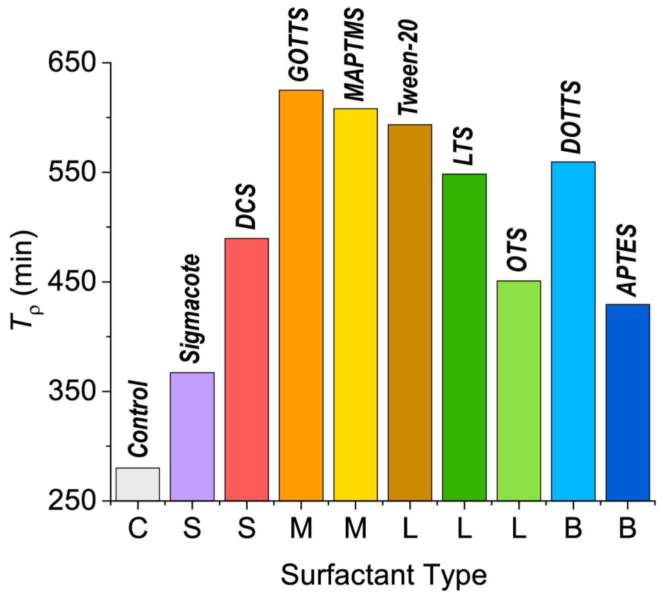

Supplemental Figure S4: pH_2 reconversion rate (T_ρ) data from Figure 6 plotted with the surfactant types grouped. The x-axis denotes the surfactant type: control (C), short (S), medium (M), long (L), and branched (B).

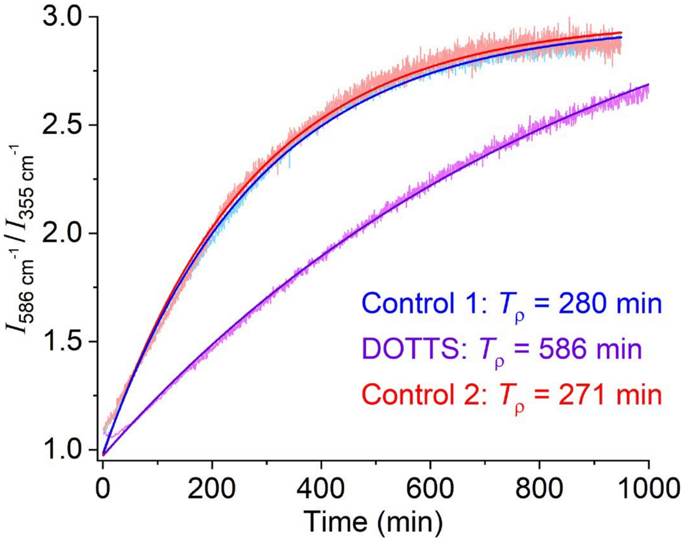

Supplemental Figure S5: Comparison of the $I_{586\ cm\text{-}1} / I_{355\ cm\text{-}1}$ time course measurements under three conditions: an NMR tube with no surfactant (Control 1; T_ρ: 280 min; blue) followed by the same NMR tube coated with DOTTS surfactant (DOTTS; T_ρ: 586 min; purple). The tube was then stripped of surfactant (following the protocol in Section 2.2 of the manuscript), and another time course measurement was completed (Control 2; T_ρ: 271 min; red). This demonstrates that adequate surfactant removal between experiments was achieved.

Acronym	Chemical Name	T_ρ (min)	Vendor	Product #
Bare	-----------	280.2 ± 1.0	---------	-----------
DCS	Dichloromethylsilane	489.5 ± 1.0	Sigma Aldrich	440248-100ML
GOTTS	(3-Glycidoxypropyl)trimethoxysilane	625.0 ± 1.4	Beantown Chemical	145300-25G
MAPTMS	3-(Methacryloyloxy)propyltrimethoxysilane	608.1 ± 1.3	Alfa Aesar	A17714
OTS	n-Octadecyltrichlorosilane	450.8 ± 0.6	Alfa Aesar	A15732
LTS	Lauryl triethoxysilane	548.4 ± 1.0	AFG Scientific	184284-1G
DOTTS	1,7-Dichloro-octamethyltetrasiloxane	559.4 ± 1.0	Sigma Aldrich	384372-25G
APTES	(3-Aminopropyl)-triethoxysilane	429.4 ± 1.0	Beantown Chemical	123580-100G
Sigmacote	Sigmacote	367.3 ± 0.6	Sigma Aldrich	SL2-25ML
Tween-80	Polyoxyethylene sorbitan monolaurate	593.5 ± 1.1	MP Biomedical	103170

Supplemental Table 1: Chemical and vendor information for surfactants used in this study, along with T_ρ tabulation (error indicative of equation fitting).
